# Impact of Empirical Antibiotic Therapy on Outcomes of Outpatient Urinary Tract Infection Due to Nonsusceptible *Enterobacterales*

**DOI:** 10.1128/spectrum.02359-21

**Published:** 2022-02-09

**Authors:** Michael W. Dunne, Sailaja Puttagunta, Steven I. Aronin, Stephen Brossette, John Murray, Vikas Gupta

**Affiliations:** a Iterum Therapeutics, Old Saybrook, Connecticut, USA; b Becton, Dickinson and Company, Franklin Lakes, New Jersey, USA; University of Texas Southwestern Medical Center

**Keywords:** urinary tract infection, antimicrobial resistance, Gram-negative bacteria

## Abstract

Resistance to oral antibiotics commonly used to treat outpatient urinary tract infections (UTIs) is increasing, but the implications of empirical treatment of resistant pathogens are not well described. Using an electronic records database, we reviewed the outcomes of patients >18 years of age who developed an outpatient UTI and had an outpatient urine culture result showing a member of the order *Enterobacterales* along with prescription data for an oral antibiotic filled on the day before, day of, or day after the culture was collected. Linear probability models were used to estimate partial effects of select clinical and demographic variables on the composite outcome. In all, 4,792 patients had 5,587 oral antibiotic prescriptions. Of 5,395 evaluable episodes, 22% of patients received an antibiotic to which the pathogen was resistant *in vitro*, and those patients were almost twice as likely to require a second prescription (34% versus 19%) or be hospitalized (15% versus 8%) within 28 days of the initial prescription fill compared to patients who received an antibiotic to which the pathogen was susceptible. Approximately 1% of *Enterobacterales* isolates were resistant to all commonly available classes of oral antibiotics. A greater risk of treatment failure was seen in patients over 60 years of age, patients with diabetes mellitus, men, and those treated with an antibiotic when prior culture identified an organism resistant to that class. The increasing resistance among members of *Enterobacterales* responsible for outpatient UTIs is limiting the effectiveness of empirical treatment with existing antibiotics, and consequently, outpatients with UTI are more likely to require additional courses of therapy or be hospitalized.

**IMPORTANCE** Resistance rates for bacteria that cause urinary tract infections (UTIs) have increased dramatically. Regional rates of resistance to commonly prescribed antibiotics now exceed 20%, which is the threshold at which the Infectious Diseases Society of America recommends therapy be guided by culture. Our goals were to describe outcomes for outpatients with UTIs caused by bacteria resistant to empirically chosen antibiotics and to create a simple stratification schema for clinicians to identify UTI patients at increased risk of treatment failure due to antibiotic mismatch. These data are relevant to clinicians, given how common uncomplicated UTIs are, and highlight the need for clinicians to understand local resistance rates and the importance of culture-guided treatment, especially in vulnerable patients. These findings also showed that 1% of bacteria were resistant to all major classes of oral antibiotics, underscoring the need for new antibiotics to treat patients with UTIs due to resistant bacteria.

## INTRODUCTION

Uncomplicated urinary tract infections (uUTIs) are among the most common bacterial infections in the outpatient setting, resulting in an estimated 15 million office or emergency department visits and 21 million prescriptions in the United States annually (1–3). Although treatment selection for acute uUTIs was straightforward in the past, management of these conditions has been complicated by reports of rising antibiotic resistance in common uropathogens (4). In a recent assessment of antimicrobial susceptibility trends observed in urinary pathogens, approximately 20% of all isolates tested were resistant to trimethoprim-sulfamethoxazole and ciprofloxacin. Resistance to nitrofurantoin, an increasingly used first-line agent for uUTIs, was reported to be 10% (5, 6).

Despite the rising rates of resistance, there are limited data on the consequences of inappropriate empirical therapy prescribed in the community for uUTI. Studies across numerous infection types clearly demonstrate the importance of “getting it right the first time.” However, it is unclear if the early appropriate therapy is as critically important among patients with less severe infections like uUTIs. To better understand the consequences of inappropriate empirical therapy among outpatients with UTIs due to Gram-negative uropathogens, we conducted a retrospective database analysis to describe rates of antibacterial resistance to commonly used oral antibiotics for uUTIs and quantify 28-day outcomes by appropriateness of empirical therapy.

(Portions of these data were previously presented at (i) Infectious Diseases Week 2018, San Francisco, CA, 3 to 7 October 2018, (ii) European Congress of Chemotherapy and Infectious Diseases, Madrid, Spain, 21 to 24 April 2018, (iii) European Congress of Chemotherapy and Infectious Diseases, Amsterdam, Netherlands, 13 to 16 April 2019, and (iv) MICROBE, American Society for Microbiology, Atlanta, GA, 7 to 11 June 2018).

## RESULTS

**FIG 1:**
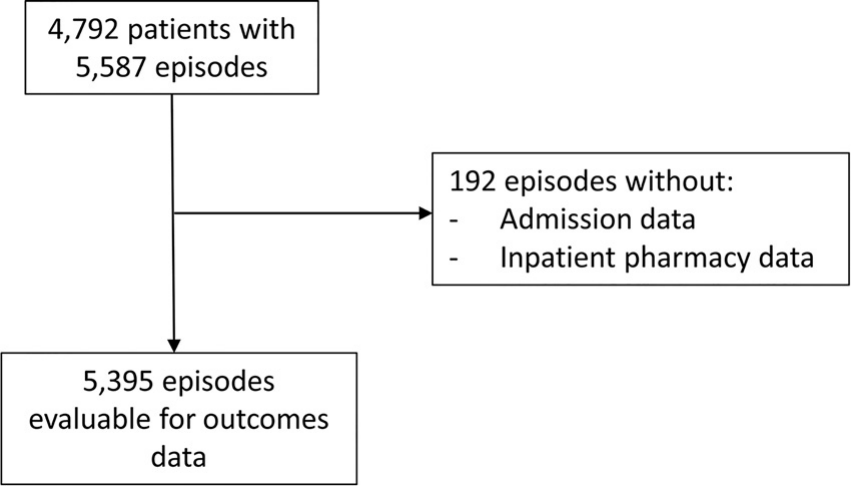
CONSORT diagram.

During the study period, 4,792 outpatients had 5,587 UTI episodes with oral antibiotic prescription data available, and 5,395 UTI episodes had antibiotic prescription data as well as admission and inpatient pharmacy data available (Fig. 1). Most patients were female, and the median age was 60.0 years. Diabetes was present in 22.5% of the population (Table 1). Quantitative culture results were measured as CFU/ml, with 3,902/5,395 (72.3%) positive urine cultures documenting levels of ≥10^5^ CFU/ml.

**TABLE 1:** Baseline patient demographics

Characteristic	Value(s) (*n* = 4,792 patients unless otherwise indicated)
Mean age ± SD (yr)	57.0 ± 22.0
Median age (yr) (range [25th, 75th percentile])	60.1 (38, 76)

Gender [no. (%)]	
Female	4,092 (85.4)
Male	700 (14.6)

% of serum creatinine measurements >2.0 mg/dL (*n* = 1,232)	1.8
% of white blood cell counts >10^5^/µL (*n* = 1,159)	9.3
No. (%) with hyperglycosuria (*n* = 3,801 measurements)	399 (10.5)
No. (%) with diabetes mellitus^*a*^	1,214 (22.5)

No. (%) of episodes (*n* = 5,395) with	
Indicated key pathogen	
Escherichia coli	4,081 (75.6)
Klebsiella spp.^*b*^	783 (14.5)
Proteus mirabilis	284 (5.3)
Other^*c*^	247 (4.6)

Indicated baseline pathogen susceptibility to prescribed antibiotic	
Susceptible	4,237 (78.5)
Nonsusceptible	1,158 (21.5)

a Diabetes mellitus was diagnosed as either a hemoglobin A1C (HbA1C) measurement of >7% or a prescription for a diabetic medication being filled in the 6 months prior to urine culture collection.

b K. pneumoniae and K. oxytoca.

c Other key pathogens include E. cloacae, E. aerogenes, C. freundii, S. marcescens, and M. morganii.

Escherichia coli was the most common pathogen (75.6%), followed by Klebsiella spp. (14.5%) and Proteus mirabilis (5.3%) (Table 2). *In vitro* resistance of these urinary tract pathogens was observed to quinolones (22.8%), β-lactams (25.1%; 6.6% were extended-spectrum β-lactamase [ESBL] positive), trimethoprim-sulfamethoxazole (27.6%), and nitrofurantoin (15.9%) (Table 3). Ciprofloxacin was the most commonly prescribed antibiotic, followed by nitrofurantoin and trimethoprim-sulfamethoxazole. Overall, 21.5% of patients received inappropriate empirical therapy (Table 1). Overall, 22.2% of patients received a subsequent prescription within 28 days, and 9.8% were hospitalized within 28 days. Of the 1,199 patients who received a subsequent prescription within 28 days, 15.8% were hospitalized in 28 days.

**TABLE 2:** Rates of represcriptions and hospitalizations within 28 days by pathogen and susceptibility

Pathogen(s)	No. (%) of episodes	No. of represcriptions/total no. of episodes (%)		% difference (95% CI)	No. of hospitalizations/total no. of episodes (%)^*b*^		% difference (95% CI)
		Susceptible	Nonsusceptible^*a*^		Susceptible	Nonsusceptible	
Overall	5,395 (100)	802/4,237 (18.9)	397/1,158 (34.3)	−15.4 (−18.4, −12)	351/4,237 (8.3)	176/1,158 (15.2)	−6.9 (−9.2, −4.8)
Escherichia coli	4,081 (75.6)	556/3,197 (17.4)	302/884 (34.2)	−16.8 (−20.2, −13.0)	218/3,197 (6.8)	120/884 (13.6)	−6.8 (−9.3, −4.5)
Klebsiella pneumoniae	733 (13.6)	148/598 (24.7)	51/135 (37.8)	−13.0 (−22.1, −4.5)	78/598 (13.0)	25/135 (18.5)	−5.5 (−13.3, 0.9)
Proteus mirabilis	284 (5.3)	45/214 (21.0)	25/70 (35.7)	−14.7 (−27.5, −2.8)	22/214 (10.3)	15/70 (21.4)	−11.1 (−22.7, −1.8)
Other^*c*^	297 (5.5)	53/228 (23.3)	20/69 (29.0)	−5.7 (−18.5, 5.4)	28/228 (12.3)	16/69 (23.2)	−10.9 (−22.8, −1.2)

a Intermediate or resistant.

b Hospitalization data were available for 5,395 UTI episodes.

c Other pathogens include E. cloacae, E. aerogenes, C. freundii, K. oxytoca, S. marcescens, and M. morganii.

**TABLE 3:** Rates of represcriptions and hospitalizations within 28 days by antibiotic received and susceptibility

Antibiotic class or resistance type	No. (%) of episodes (*n* = 5,395)	% (no.) of episodes with nonsusceptible isolates (*n* = 5,395)	No. of represcriptions/total no. of episodes (%)		% difference (95% CI)	No. of hospitalizations/total no. of episodes (%)^*b*^		% difference (95% CI)
			Susceptible	Nonsusceptible^*a*^		Susceptible	Nonsusceptible	
Fluoroquinolone	1,873 (34.7)	22.8 (1,232)	237/1,483 (16.0)	140/390 (35.9)	−19.9 (−25.1, −15.0)	130/1,483 (8.8)	65/390 (16.7)	−7.9 (−12.2, −4.2)
β-Lactam^*c*^	1,309 (24.3)	25.1 (329)						
ESBL^*d*^		6.6 (356)	224/980 (22.9)	91/329 (27.7)	−4.8 (−10.5, 0.5)	81/980 (8.3)	48/329 (14.6)	−6.3 (−10.8, −2.4)
Trimethoprim-sulfamethoxazole	1,041 (19.3)	27.6 (1,491)	134/753 (17.8)	106/288 (36.8)	−19.0 (−25.3, −13)	71/753 (9.4)	45/288 (15.6)	−6.2 (−11.2, −1.8)
Nitrofurantoin	1,228 (22.8)	15.9 (857)	214/1,055 (20.3)	64/173 (37.0)	−16.7 (−24.5, −9.4)	73/1,055 (6.9)	23/173 (13.3)	−6.4 (−12.4, −1.8)
Fosfomycin	NA^*e*^	NA	0/1 (0.0)			0/1 (0.0)		

a Intermediate or resistant.

b Hospitalization data were available for 5,395 UTI episodes.

c The number of episodes nonsusceptible to a β-lactam out of those that with an initial β-lactam prescription filled was 329 out of 1,309.

d Isolates were confirmed as ESBL positive by commercial panels or were determined to be intermediate/resistant to an extended-spectrum cephalosporins (ceftriaxone, cefotaxime, ceftazidime, or cefepime).

e NA, not applicable.

Treatment failure occurred in 34.3% of patients who received inappropriate empirical therapy, compared to 18.9% in patients who received appropriate empirical therapy. Similarly, the rates of hospitalization were higher for patients who were treated with an antibiotic to which the pathogen was nonsusceptible (15.2% versus 8.3% for nonsusceptible and susceptible pathogens, respectively) (Table 2). The 28-day antibiotic refill rate did not differ significantly by baseline pathogen or initial antibiotic prescribed (Table 3). A threshold of ≥10^5^ CFU/ml on the index urine culture did not distinguish between the need for a second prescription or hospitalization within 28 days relative to an organism burden of <10^5^ CFU/ml. Diabetics were more likely to have an infection with E. coli (*P* < 0.001) and more likely to be treated with an antibiotic to which the E. coli isolate was not susceptible (*P* < 0.001).

Of the 5,395 outpatient *Enterobacterales* isolates, 32% were pansusceptible (Table 4), nearly 7% were ESBL-producing strains (Table 3), and almost 1% (47/5,395) were resistant to all classes of oral antibiotics commonly available in the United States (Table 4). The risk of treatment failure tended to increase with the number of classes of antibiotics to which the pathogen was resistant.

**TABLE 4:** Rates of represcriptions and hospitalizations within 28 days by degree of antibiotic class resistance

Degree of resistance	Total no. of episodes (%)	Represcriptions		*P* value^*e*^	Hospital admissions		*P* value
		No.	%		No.	%	
Overall^*a*^	5,395 (100)	1,199	22.2		527	9.8	
Pansusceptible	1,725 (32.0)	279	16.2	Index	125	7.2	Index
Resistant to^*b*^							
1 or 2 classes	3,478 (64.5)	866	24.9	<0.0001	350	10.1	<0.0001
3 classes^*c*^	145 (2.7)	43	29.7	<0.0001	35	24.1	<0.0001
4 classes^*d*^	47 (0.9)	11	23.4	0.1863	17	36.1	<0.0001
3 or 4 classes	192 (3.6)	54	28.1	<0.0001	52	27.1	<0.0001

a Prescription and hospitalization data were available for 5,395 UTI episodes in 4,792 patients; this includes all UTI episodes regardless of colony count of baseline pathogen.

b Groupings of classes above are mutually exclusive.

c Resistance to quinolones, trimethoprim-sulfamethoxazole, and β-lactams.

d Resistance to 4 classes also includes resistance to nitrofurantoin.

e *P* values were determined by the Cochran-Mantel-Haenszel test.

Demographic and clinical variables that were associated with an increased risk of treatment failure include increasing age over 60 years, diabetes mellitus, male gender (by definition, UTI in men is classified as complicated), and treatment with a quinolone, nitrofurantoin, or β-lactam when a prior culture had identified an organism resistant to that same class. The baseline risk of treatment failure for urinary *Enterobacterales* in a 60-year-old woman with normal creatinine, no diabetes, and no history of resistant *Enterobacterales*, treated with a quinolone, was 17%. That risk of treatment failure increased incrementally as additional risk factors were identified (Table 5).

**TABLE 5:** Linear probability model estimates of partial effects on treatment failure^*a*^

Variable	Partial effect (%)	*P* value^*b*^
Age, per decade, over 60 yrs	2	<0.01
Male	6	<0.01
Diabetes mellitus	6	0.02
Elevated creatinine (>2 mg/dL)	11	0.1 (n.s.)

Index treatment with AND previous resistance to the same class		
Quinolone	22	<0.01
Trimethoprim-sulfamethoxazole	26	<0.01
Nitrofurantoin	36	<0.01

Index treatment (vs quinolone)		
Amoxicillin	21	<0.01
Augmentin	7	0.02
Nitrofurantoin	6	<0.01
Trimethoprim-sulfamethoxazole	8	<0.01
Cephalexin	5	0.03

Baseline risk of failure^*c*^	17	<0.001

a Covariates in model estimates were age, sex, prior ESBL, prior quinolone nonsusceptibility (NS), prior trimethoprim-sulfamethoxazole NS, prior nitrofurantoin NS, index treatment (quinolone base level), index treatment by prior quinolone NS, index treatment by prior trimethoprim-sulfamethoxazole NS, index treatment by prior nitrofurantoin NS, and site-level fixed effects.

b *P* values were determined by the Cochran-Mantel-Haenszel test. n.s., not significant.

c Constant: 60 year-old, female, empirical quinolone treatment, no history of resistant pathogen, no laboratory data prior to treatment.

## DISCUSSION

In this cohort of over 5,000 patients at 15 institutions in the United States, significant *in vitro* resistance was identified across the antibiotics commonly used to treat community urinary tract infections, exceeding 20% for quinolones, β-lactams, and trimethoprim-sulfamethoxazole. As a consequence, 22% of patients in this study did not initially receive an antibiotic that was effective against the offending pathogen *in vitro*.

The within-28-days antibiotic represcription rate for patients whose initial antibiotic did not match the susceptibility of the identified pathogen was approximately 34%, almost twice the rate for patients with susceptible pathogens, and did not differ significantly by baseline pathogen, CFU count at baseline, or the initial antibiotic prescribed. A similar observation was made in a small retrospective study of UTI patients treated in the emergency department with cephalexin, where resistance rates were as high as 45% and return visits for those who received mismatched therapy were more than double (7). These data confirm the role of antibiotic therapy in the treatment of UTI, as well as the importance of an initial treatment tailored to the offending pathogen.

In 2016, 9.2% of the U.S. population was diagnosed with diabetes mellitus (8). Relative to the national prevalence rate, in this population of patients with urinary tract infection, diabetics were significantly overrepresented, with a rate 2.5 times the national average. Diabetes has previously been found to be associated with a higher risk for UTI, including higher frequencies of recurrent episodes, represcriptions, and hospitalizations (9–11). In some circumstances, diabetic women with a UTI are considered to have a complicated infection (12), and the data from these analyses would support that observation. In this study, diabetics were more likely to present with E. coli and the E. coli isolates were more likely to be nonsusceptible to the prescribed antibiotic. A predisposition for diabetics to fail treatment may relate to an underlying immune dysfunction, although repeat treatment courses and subsequent selection of resistant pathogens may be just as important.

The parameter estimates from the linear probability models (LPMs) provide a patient-centered risk adjustment index that may help identify patients who are at significantly higher risk of treatment failure and for whom more effective initial antibacterial regimens would be advantageous. Among the risk factors for the composite failure event, empirical treatment with an antibiotic class for which there is a history of a resistant pathogen is most significant. For example, a 70-year-old woman with diabetes mellitus and a history of a quinolone-resistant organism who is treated with a quinolone has an estimated probability of failure of 47%, far exceeding the baseline risk of failure of 17%. In such a patient, a high level of confidence in the *in vitro* activity of the empirically chosen antibiotic based on the local antibiogram would be especially important.

The 2011 treatment guidelines of the Infectious Diseases Society of America (IDSA) for uncomplicated urinary tract infection (13) recommend that empirical treatment regimens be selected based on an expected resistance rate of <20% in the community to avoid the potential for a poor outcome associated with inappropriate therapy. In 2021, this 20% threshold has been exceeded for β-lactams, trimethoprim-sulfamethoxazole, and quinolones, as well as nitrofurantoin for *Enterobacterales* other than E. coli. The remaining FDA-approved agent for uUTI in the treatment guidelines is fosfomycin, which is rarely prescribed, due in part to questionable efficacy (14) and the inability for most clinical laboratories to perform susceptibility testing for this agent.

This study found that 22% of patients for whom a baseline urine culture was sent were failing initial empirical antibiotic treatment for uUTI, a rate that is nearly twice as high as for those who receive appropriate empirical antibiotics. This implies that, in the United States, commonly prescribed empirical antibiotics, as recommended in IDSA’s treatment guidelines, are no longer capable of achieving their stated objective. Waiting for urine culture results before prescribing therapy is a possible solution, but this approach may be less effective, more costly (15), and ultimately, an unviable strategy. Besides prolonging the suffering of women with uUTI, this approach may increase the likelihood of progression to upper tract disease (16) and, as recently reported from England, may result in higher mortality rates in elderly patients (17). Either new oral antibiotics more likely to cover the offending pathogens need to become available, especially important in patients at higher risk of infection and treatment failure, or the promise of rapid diagnostic tests in which the genus and species of the pathogen and its relevant resistance mutations are available to guide empirical treatment needs to be realized.

These analyses have some limitations. Patients included in the analysis had a baseline urine culture sent, which may reflect baseline patient characteristics differentiating them from patients who had no culture sent. The patients presented at a clinic associated with a hospital microbiology laboratory and may not reflect results from a freestanding clinic. While there may have been subtle differences in the methods of determining susceptibility used by the different testing sites, these differences were likely not significant, as the outcomes were consistent with other published epidemiologic studies. Due to coding limitations, other comorbidities that could have affected the LPMs could not be assessed, and omitted variables correlated with those in the model may bias model estimates. To account for the fact that symptomatology to justify a switch in antibiotic therapy was not available, we purposely excluded antibiotic switches within 1 day of the availability of culture results, which may have occurred simply because the initial regimen was adjusted after culture outcome and not because of ongoing symptoms. We cannot, however, be certain that our approach completely removed this bias. For any reason, however, the environmental effects of multiple courses of different classes of antibiotics for the same episode of infection should not be ignored. The hospitalization outcomes were not based on international classification of diseases (ICD) codes, and as a consequence, the hospitalization rates may not reflect the true rates in these communities. The definition of diabetes mellitus was conservative and limited to concomitant ambulatory prescription fills of medications to control diabetes and HbA1C measurements, although the increased proportion of patients with diabetes is consistent with other published surveys (9, 18). Lastly, the sample size may have limited the power of the study to draw certain conclusions; elevated creatinine and hyperglycosuria were associated with treatment failure in other studies but were not identified here.

Increasing rates of resistance among the *Enterobacterales* isolates responsible for outpatient urinary tract infection are limiting the effectiveness of empirical treatment with existing antibiotics. As a consequence, outpatients, including women with uUTI, are more likely to experience a prolonged course of infection. For as many as 1% of women, or approximately 200,000 episodes every year in the United States alone, there is no commonly available oral antibiotic to treat their infection; many of these women will require intravenous therapy. In order for guidelines to continue to support empirical antibiotic therapy for UTI, there will need to be either point-of-care rapid diagnostics that identify both species and resistance determinants or new oral antibiotics effective against resistant uropathogens.

## MATERIALS AND METHODS

### Study population.

Patients from 15 institutions in the U.S. BD Insights Research Database (Becton, Dickinson and Company, Franklin Lakes, NJ, USA) with outpatient urine culture results, demographic information, and prescription data collected between 2015 and 2017 were considered for inclusion in this study. Patients were included if they (i) had a quantitative urine culture positive for a member of the order *Enterobacterales*, including E. coli, Klebsiella pneumoniae, Klebsiella oxytoca, Enterobacter aerogenes, Enterobacter cloacae, Serratia marcescens, Citrobacter freundii, Proteus mirabilis, or Morganella morganii, and (ii) received a prescription for any of the following oral antibiotics on the day prior to the urine culture collection date, the index urine culture date, or the day after a urine culture index date: fluoroquinolones, trimethoprim-sulfamethoxazole, nitrofurantoin, fosfomycin, and β-lactams (amoxicillin, amoxicillin/clavulanate, or oral cephalosporins). For patients with multiple urine cultures within the 28-day follow-up period, only the first episode with a corresponding antibiotic prescription was included.

### Data elements and outcomes.

Demographic elements included age at index date, gender, antibiotic therapy, quantitative urine culture, and susceptibility results. Laboratory data (i.e., white blood cell count, hemoglobin, creatinine, and urine glucose) within 7 days of the index urine culture were evaluated, when available. A patient was considered to have a diagnosis of diabetes mellitus if the patient had either a hemoglobin A1C (HbA1C) measurement of >7% or a prescription filled for a diabetic medication in the 6 months prior to urine culture collection. We also evaluated the presence of urine glucose in episodes that had a urinalysis done on the day of index culture collection as an additional possible surrogate for uncontrolled blood sugar. The CFU/mL from the quantitative urine culture was documented. Pathogens were considered susceptible or nonsusceptible to the antimicrobial class based on susceptibility testing performed at the local institution.

Empirical antibiotic therapy was defined as appropriate if the patient received a prescription for an antibiotic with *in vitro* activity (defined as “susceptible” in the database) against all identified *Enterobacterales* isolates on the index urine culture date ± 1 day. The primary outcome of interest was antibiotic treatment failure. Antibiotic treatment failure was defined as (i) receipt of a subsequent antibiotic prescription within 28 days of the initial prescription or (ii) a UTI-related hospital admission within 28 days of the initial prescription. When evaluating subsequent prescriptions within 28 days, prescriptions within 1 day of the availability of antimicrobial susceptibility test results were excluded to avoid including antimicrobial modifications by the clinician simply due to the availability of susceptibility results. Both all-cause and UTI-related 28-day hospital admissions were examined. Hospital admissions were considered UTI related if the patient received antimicrobial therapy (intravenous or oral) within the first 3 days and had a positive urine culture.

### Statistical analysis plan.

Two models were estimated, one for all episodes and one for episodes that had general laboratory data within 7 days of the index culture. Model parameters were estimated by ordinary least-squares with robust standard errors (Stata version 14; Stata Corp LP). The estimated models are linear probability models (LPMs) that tested for statistical significance of estimated coefficients (*P* < 0.01). Hospital-level fixed effects were included to control for any persistent differences between hospital and clinic policies, procedures, laboratory practices, and populations served. The model for episodes with general laboratory examination was additionally controlled for abnormal HbA1C (>7%), creatinine (>2 mg/dL), and white blood cell count (>10 × 10^9/L) measurements.
