# Supplementary material for: Impact of Empirical Antibiotic Therapy on Outcomes of Outpatient Urinary Tract Infection Due to Nonsusceptible Enterobacterales
Source: Microbiol Spectr. 2022 Feb 9;10(1):e02359-21. doi: 10.1128/spectrum.02359-21 (PMC8826825; doi:10.1128/spectrum.02359-21)
Supplement: SUPPLEMENTAL FILE 1 — Supplemental material. Download SPECTRUM02359-21_Supp_1_seq4.pdf, PDF file, 0.1 MB [file spectrum02359-21_supp_1_seq4.pdf]

Supplemental Table 1. 28-day Represcription and hospitalization rates by pathogen an antibiotic received

**Antibiotic class** / Pathogen	Frequency (%)	Non-susceptible to class N = 5395 % (n)	28-day Represcription n/N (%)		Hospitalizations[b] n/N (%)	
			Susceptible	Non-susceptible[a]	Susceptible	Non-susceptible
**All Empiric Treatments Received**						
**Overall**			**802/4237 (18.9)**	**397/1158 (34.3)**	**351/4237 (8.3)**	**176/1158 (15.2)**
Escherichia coli	4,081 (75.6%)		556/3197 (17.4%)	302/884 (34.2%)	218/3107 (7.0%)	120/884 (13.6%)
Klebsiella pneumoniae	733 (13.6%)		148/598 (24.7%)	51/135 (37.8%)	78/598 (13.0%)	25/135 (18.5%)
Proteus mirabilis	284 (5.3%)		45/214 21.0%)	25/70 (35.7%)	22/214 (10.3%)	15/70 (21.4%)
Other[c]	297 (5.5%)		53/228 (23.3%)	20/69 (29.0%)	28/228 (12.3%)	16/69 (23.2%)
**Fluoroquinolone**						
**Overall**	**100.0**	**22.8 (1232)**	**237/1483 (16.0)**	**140/390 (35.9)**	**130/1483 (8.8)**	**65/390 (16.7)**

Escherichia coli	70.7	27.2 (1,111)	126/979 (12.9)	128/345 (37.1)	59/979 (6.0)	53/345 (15.4)
Klebsiella pneumoniae	16.2	6.8 (50)	68/288 (23.6)	3/16 (18.8)	40/288 (13.9)	3/16 (18.8)
Proteus mirabilis	5.5	18.7 (53)	12/83 (14.5)	7/20 (35.0)	12/83 (14.5)	6/20 (30.0)
Other[c]	7.6	6.1 (18)	31/133 (23.3)	2/9 (22.2)	19/133 (14.3)	3/9 (33.3)
**ESBL+/β-lactam**						
**ESBL+**	**100.0**	**6.6 (356)**	**224/980 (22.9)**	**91/329 (27.7)**	**81/980 (8.3)**	**48/329 (14.6)**
**β-lactam**		**329/1309 [25.1]**				
Escherichia coli	76.1	7.4 (303)	152/737 (20.6)	71/259 (27.4)	46/737 (6.2)	28/259 (10.8)
Klebsiella pneumoniae	13.6	6.0 (44)	48/149 (32.2)	11/29 (37.9)	22/149 (14.8)	8/29 (27.6)
Proteus mirabilis	7.0	2.5 (7)	21/79 (26.6)	2/12 (16.7)	11/79 (13.9)	3/12 (25.0)
Other	3.4	0.7 (2)	3/15 (20.0)	7/29 (24.1)	2/15 (13.3)	9/29 (31.0)
**Trimethoprim-sulfamethoxazole**						
**Overall**	**100.0**	**27.6 (1491)**	**134/753 (17.8)**	**106/288 (36.8)**	**71/753 (9.4)**	**45/288 (15.6)**

Escherichia coli	76.9	32.6 (1,330)	87/536 (16.2)	98/265 (37.0)	47/536 (8.8)	39/265 (14.7)
Klebsiella pneumoniae	11.9	12.6 (92)	21/112 (18.8)	5/12 (41.7)	13/112 (11.6)	2/12 (16.7)
Proteus mirabilis	6.0	15.8 (45)	12/53 (22.6)	2/9 (22.2)	4/53 (7.5)	3/9 (33.3)
Other	5.2	8.1 (24)	14/52 (26.9)	1/2 (50.0)	7/52 (13.5)	1/2 (50.0)
**Nitrofurantoin**						
**Overall**	**100.0**	**15.9 (857)**	**214/1055 (20.3)**	**64/173 (37.0)**	**73/1055 (6.9)**	**23/173 (13.3)**
Escherichia coli	81.7	3.4 (138)	197/971 (20.3)	9/32 (28.1)	66/971 (6.8)	5/32 (15.6)
Klebsiella pneumoniae	10.9	58.3 (427)	12/55 (21.8)	32/79 (40.5)	6/55 (10.9)	12/79 (15.2)
Proteus mirabilis	2.6	53.5 (152)	-	13/32 (40.6)	-	3/32 (9.4)
Other	4.8	47.1 (140)	5/29 (17.2)	10/30 (33.3)	1/29 (3.4)	3/30 (10.0)
**Fosfomycin**						
**Overall**	**N/A**	**N/A**	**0/1 (0.0)**	**-**	**0/1 (0.0)**	**-**
Klebsiella pneumoniae	N/A	N/A	0/1 (0.0)	-	0/1 (0.0)	-
